# Obituary

**Published:** 1893-10

**Authors:** 


### Obituary.

Died September 3, 1893, Dr. W. C. Wardlaw, of Augusta, Ga.

Dr. Wardlaw had been in feeble health for sometime, though it was not generally known to the profession. His demise will be a great surprise and a matter of sorrow to his very large circle of friends and acquaintances; hardly any dental practitioner of the South was more generally and more favorably known than Dr. Wardlaw. He was a man of broad attainments, one who stood well in the esteem of all who knew him. The breach occasioned by his death will not soon be filled. His family will have the sincere sympathy of a large circle of friends.

